# InfectionCMA: A Cell MicroArray Approach for Efficient Biomarker Screening in In Vitro Infection Assays

**DOI:** 10.3390/pathogens11030313

**Published:** 2022-03-03

**Authors:** Ana C. Magalhães, Sara Ricardo, Ana C. Moreira, Mariana Nunes, Margarida Tavares, Ricardo J. Pinto, Maria Salomé Gomes, Luisa Pereira

**Affiliations:** 1 i3S–Instituto de Investigação e Inovação em Saúde, Universidade do Porto, 4200-135 Porto, Portugal; acmagalhaes@ipatimup.pt (A.C.M.); sricardo@ipatimup.pt (S.R.); ana.s.moreira@ibmc.up.pt (A.C.M.); mnunes@ipatimup.pt (M.N.); rjpinto@ipatimup.pt (R.J.P.); sgomes@ibmc.up.pt (M.S.G.); 2 Ipatimup–Instituto de Patologia e Imunologia Molecular, Universidade do Porto, 4200-135 Porto, Portugal; 3 ICBAS–Instituto de Ciências Biomédicas Abel Salazar, Universidade do Porto, 4050-313 Porto, Portugal; 4 TOXRUN, Toxicology Research Unit, University Institute of Health Sciences, Advanced Polytechnic and University Cooperative (CESPU), 4585-116 Gandra, Portugal; 5 IBMC–Instituto de Biologia Molecular e Celular, Universidade do Porto, 4200-319 Porto, Portugal; 6 Department of Infectious Diseases and Emerging Infectious Disease Unit, CHUSJ–Centro Hospitalar Universitário S. João, 4200-319 Porto, Portugal; margarida.tavares@chsj.min-saude.pt; 7 Public Health and Forensic Sciences and Medical Education Department, FMUP–Faculdade de Medicina, Universidade do Porto, 4200-319 Porto, Portugal; 8 EPIUnit–Instituto de Saúde Pública, Universidade do Porto, 4050-091 Porto, Portugal

**Keywords:** in vitro infectious assays, cell microarrays, immunocytochemistry, SARS-CoV-2, human receptors for viruses, cell cycle and apoptosis

## Abstract

The recently emerged severe acute respiratory syndrome coronavirus 2 (SARS-CoV-2) has forced the scientific community to acquire knowledge in real-time, when total lockdowns and the interruption of flights severely limited access to reagents as the global pandemic became established. This unique reality made researchers aware of the importance of designing efficient in vitro set-ups to evaluate infectious kinetics. Here, we propose a histology-based method to evaluate infection kinetics grounded in cell microarray (CMA) construction, immunocytochemistry and in situ hybridization techniques. We demonstrate that the chip-like organization of the InfectionCMA has several advantages, allowing side-by-side comparisons between diverse cell lines, infection time points, and biomarker expression and cytolocalization evaluation in the same slide. In addition, this methodology has the potential to be easily adapted for drug screening.

## 1. Introduction

In vitro modelling is one of the first research steps in the field of infectious diseases, allowing scientists to investigate, amongst other things, which host cell types are permissive to infection, which molecular mechanisms govern the entrance of pathogens into cells and their replication within, and what alterations are induced in the host cell metabolism and viability due to infection [1,2,3]. This modelling is also the first step in the preclinical evaluation of novel or repurposed drugs and compounds, saving precious time and money at the in vivo testing and clinical trial levels [1,4,5]. The value of in vitro modelling justifies the various efforts being made in improving settings, aiming to better mimic the complex biological systems, such as, for example, recurring stem-cell differentiation, establishing co-culture assays, and implementing three-dimensional organoids [6]. The global coronavirus disease 2019 (COVID-19) pandemic is the perfect illustrative example of the vital importance of in vitro modelling as a research and translational tool. COVID-19 is a viral respiratory disease that newly emerged by the end of 2019 in Wuhan, China, which rapidly spread worldwide. By the end of the year 2021, conservative estimates point to 290 million SARS-CoV-2 infections and 5.4 million related deaths globally (https://coronavirus.jhu.edu/map.html, accessed on 31 December 2021). Meanwhile, in vitro modelling allowed scientists to elucidate several aspects of the SARS-CoV-2 cycle in the host cells [7,8,9,10,11,12,13,14,15,16,17,18,19,20,21,22,23,24,25,26]. The viral spike protein binds to the human receptor ACE2 and is cleaved by the transmembrane serine protease 2 (TMPRSS2), allowing the fusion of the viral and host membranes for entrance into the cell. The translation of the viral RNA into non-structural proteins suppresses the translation of host mRNAs in favor of the viral mRNAs for the production of virions. Further transformations of the host cells include the endoplasmic reticulum being modified into double-membrane vesicles, where more viral RNA can be replicated and translated. The exit of the new virus particles occurs via Golgi and/or lysosomes, and at the same time the host furin protein cuts the spike protein, preparing the virus to infect another cell. In vitro modelling has also been key for preliminary drug screenings [27,28,29] of FDA-approved drugs, leading to the identification of several compounds that show in vitro antiviral properties against SARS-CoV-2. 

The pressure regarding acquiring real-time knowledge on SARS-CoV-2 infection, and the huge restrictions in access to reagents in the beginning of the pandemic, also demonstrated how essential it is to design efficient in vitro set-ups. Simple solutions can be available in a specific research field, but they do not often cross to other fields. This is the case of a simple automation technique used in the field of histology, the CMA. Cultured cells, namely fixed infected cells, can be included in paraffin-embedded blocks similarly to the formalin-fixed paraffin-embedded (FFPE) tissues from surgical pathology specimens (reviewed in [30]). Then, CMAs can be created, consisting of several tens to hundreds of cores from one or various individual cell blocks, integrated into a microarray form, on a single or various slides. CMAs allow various ancillary studies to be performed, such as the rapid and high-throughput analysis of molecular targets through immunocytochemistry (ICC) [31,32] and in situ hybridization (ISH) [33]. The CMA block can be easily archived for retrospective studies [34]. Additionally, the ICC processing can be further automatized by the integration of microfluidic techniques [35], enabling further reductions in reagent quantities and process time, the precise control of assay conditions, and ease of multiplexing.

In this work, we want to demonstrate to the infectious disease research community the advantages of the CMA. We call this CMA design InfectionCMA due to its ability to model infection kinetics. We demonstrate the practicability and efficiency of the InfectionCMA compared to traditional in vitro infection assays in two SARS-CoV-2 investigations, with the first aiming to compare the effect of viral load in infection kinetics on Vero cells, and the second aiming to compare the infection kinetics between human cell lines and Vero cells. Although the establishment of the InfectionCMA implies additional preparation time and reagents, this extensively compensates for the more complex, laborious and reagent-consuming infectious assays in biosafety level 3 (BSL3) conditions, ICC preparations and microscopic observations needed to obtain the same output through a traditional in vitro infection assay.

## 2. Materials and Methods

### 2.1. Cell Lines

Vero cells from the kidney of African green monkey (ATCC, #CCL-81; ATCC, Manassas, VA, USA), Caco-2 cells from human primary colorectal adenocarcinoma (in-house cell bank) and Huh-7 cells from human hepatocellular carcinoma (kindly gifted by Tiago Duarte) were used in this work after confirmation of a mycoplasma-negative status. The cell lines were propagated in Dulbecco’s modified Eagle medium (DMEM, Thermo Scientific, Waltham, MA, USA) supplemented with 10% fetal bovine serum (FBS, Labclinics, Barcelona, Spain), 100 U/mL of penicillin, and 100 ug/mL of streptomycin. Cells were maintained at 37 °C, under a 5% CO_2_ humidified atmosphere.

### 2.2. Isolation of the SARS-CoV-2

SARS-CoV-2 was isolated from the nasopharyngeal sample of a hospitalized symptomatic patient from North Portugal in the spring of 2020. The molecular diagnosis was confirmed by RT-PCR for three SARS-CoV-2 genes (ORF1ab, E and N–Fosun COVID-19 RT-PCR Detection Kit; Fosun Pharma USA Inc., Princeton, NJ, USA). The nasopharyngeal sample was preserved at −80 °C in the hospital, until being transferred to our lab (distance of 1 km from the hospital) 15 days later, for immediate isolation of the virus.

The isolation was performed by inoculation in Vero cells, and the resultant supernatants were filtered and used as virus stocks preserved at −80 °C. Titers were determined by the focus forming assay (FFA). Briefly, ten-fold dilutions of viral supernatants were inoculated in Vero cells, seeded 24 h prior to infection in 96-well plates. After 1 h of incubation, an overlay medium of DMEM with 4% FBS and 3% carboxymethyl cellulose (CMC) in a ratio of 1:1 was added to the inoculum, and the infected monolayer was incubated for 48 h at 37 °C in 5% CO_2_. Cells were fixed with 4% paraformaldehyde at room temperature (RT), permeabilized with 0.2% Triton-X 100 (Sigma, St. Louis, MO, USA) for 10 min, blocked with 1% bovine serum albumin (BSA–Sigma, St. Louis, MO, USA) for 10 min, and incubated with a mouse SARS-CoV-2 spike antibody (GTX632604, GeneTex, Irvine, CA, USA) for 1 h at RT. Afterwards, cells were incubated with anti-mouse Alexa 488-conjugated secondary antibody for 1 h (Thermo Scientific, Waltham, MA, USA), followed by nuclear staining with 4′,6-diamidino-2-phenylindole (DAPI–BioRad, Hercules, CA, USA) for 10 min, before analysis. Images of each well were acquired on the IN Cell Analyzer 2000 (Cytiva, Marlborough, MA, USA), using an appropriate magnifying objective, and the fluorescent focus units (FFU) were counted.

All experiments involving infectious SARS-CoV-2 were performed in BSL3 conditions in accordance with institutional guidelines.

### 2.3. Viral Whole Genome Sequencing

The viral whole-genome sequencing was performed on extracted RNA from the isolated virus. Briefly, this protocol [36,37] consisted of: reverse-transcription with the SuperScriptTM VILOTM cDNA synthesis Kit (Thermo Fisher Scientific, Waltham, MA, USA); PCR enrichment of the SARS-CoV-2 genome and five human gene expression controls with the Ion AmpliSeqTM SARS-CoV-2 Research Panel; library construction with the Ion AmpliSeqTM Library Kit Plus; library quantification and size range verification at the 2200 TapeStation Automated Electrophoresis System, using the High Sensitivity DNA ScreenTape (Agilent Technologies, Santa Clara, CA, USA); next-generation sequencing (NGS) on the Ion S5XL system with the Ion 530™ chip; and raw data extracted with the Ion Torrent pipeline.

The bioinformatic pipeline consisted of: alignment of the raw data versus the reference genome (severe acute respiratory syndrome coronavirus 2 isolate Wuhan-Hu-1; accession number NC_045512.2; [38]) with BWA tool; variant calling with three tools, FreeBayes, BCFtools and GATK, with editing of variants identified in at least two; variant annotation with SnpEff; consensus sequence was inferred with Bcftools.

### 2.4. In Vitro Infection Assays

For the CMA construction, cells were seeded 24 h prior to infection in T-25 flasks at a density of 7 × 10^5^/flask. On the infection day, the cell culture medium in each flask was discarded and cell monolayers were inoculated with SARS-CoV-2 (diluted appropriately in serum free DMEM) with a multiplicity of infection (MOI) of 0.01 and 0.001 for the first CMA construction test with Vero cells only, and an MOI of 1 for the remaining experiments with the human and Vero cell lines. Viral infection was allowed to proceed for 1 h at 37 °C in 5% CO_2_, and after that, DMEM with 2% of FBS was added to the flasks. Cells were collected at various time points: for the first InfectionCMA at 4 h, 24 h, 48 h and 72 h; and for the second InfectionCMA at 16 h, 36 h and 72 h.

The proteins and RNA for the Western blotting and qRT-PCR were obtained by conducting three independent infection assays in 6-well plates, with a cell density of 1 × 10^5^/well, following the same conditions as described above. Cells were collected at 16 h, 48 h and 96 h.

In order to confirm that infectious viral particles were produced after the infection assays, the viral yields were determined by FFA, as described in Section 2.1, at each observation-point.

### 2.5. InfectionCMA Construction

The establishment of the InfectionCMA followed the published protocols [32]. For the various time point controls and infected cell cultures, before fixation, the medium/supernatant was removed and cells were washed with PBS 1×, thus discarding remnants of medium and non-internalized viruses. Then, cells were independently collected by scraping them from the flask with PBS 1×, followed by centrifugation and fixation with 10% neutral-buffered formalin. After fixation, each cell pellet was embedded in HistoGel (Thermo Scientific, Waltham, MA, USA) according to the manufacturer’s instructions, followed by standard histological processing and paraffin embedding. Each cell block (donor block) was sectioned, and hematoxylin and eosin (H&E) stained for morphology control. Microscopic visualization of these H&E slides guided the selection of cell-rich sections for punching the 1.5 mm diameter cores. For CMA arrangement, we organized the cell lines (controls and infected) per lines, and the time points after infection per columns, or the other way around depending on whether we had more cell lines or infection time points. Then, the cores from each donor block were collected and transported to the previously defined position in the recipient paraffin block. The recipient block used had 35 cores with 1.5 mm diameter (5 lines × 7 columns) and was constructed with liquid paraffin and microarray molds. If the cores exceeded the number of samples, the empty ones were filled with paraffin. After construction, the InfectionCMAs were homogenized at 37 °C overnight and sectioned with a standard microtome (3–4 µm thickness) into slides.

### 2.6. Immunocytochemistry

After deparaffinization, heat-induced (98 °C) antigen retrieval was performed with a citrate buffer (pH 6.0) or EDTA buffer, and slides were incubated with hydrogen peroxide 3%. The InfectionCMAs were immunostained with the following primary antibodies: SARS-CoV-2 spike [1A9] (mouse; GTX632604, Genetex, Irvine, CA, USA), SARS-CoV-2 nucleocapsid [6H3] (mouse; GTX632269, Genetex, Irvine, CA, USA), ACE2 (mouse; MA5-31395, Thermo Scientific, Waltham, MA, USA), Neuropilin 1 [EPR3113] (rabbit; ab183495, Abcam, Cambridge, UK), Ki-67 (rabbit; MA5–14520, Thermo Scientific, Waltham, MA, USA) and BCL2 (rabbit; AB196495; Abcam, Cambridge, UK). Primary antibodies were detected using a secondary antibody with horseradish peroxidase (HRP) polymer (Dako–Agilent, Santa Clara, CA, USA) and visualization of the reaction was performed using diaminobenzidine (DAB) according to the manufacturer’s instructions. The DAB brown coloration represents the positive staining, and the hematoxylin (blue coloration) was used as nuclear counterstain, dehydrated, and mounted. We confirmed that the tested human proteins were expressed in the tissues (from kidney and liver) represented by the used monkey and human cell lines (Supplementary Figure S1), while the viral ones were not expressed (confirming the SARS-CoV-2 specificity, as tissues were collected before the pandemic).

ICC images were evaluated in an Olympus Cx31 microscope (Olympus Lifesciences, Tokyo, Japan) by two independent observers (ACMagalhães and SR). The cytolocalization (nuclear, cytoplasm or membrane) of the staining was identified, and the relative percentage of the stained cells (in the entire core field) was registered in the following categories: 0–1%, >1–10%, >10–25%, >25–50%, >50–75%, and >75%. Digitalization of the InfectionCMA slides was obtained in NanoZoomer 2.0 HT (Hamamatsu Photonics, Hamamatsu City, Japan) with a magnification of 40×, and the high-resolution digital images were visualized in the associated NDP.view2 software in order to select representative figures.

### 2.7. mRNA In Situ Hybridization Assay

The mRNA ISH was performed using the RNAScope^®^ Probe-V-nCoV2019-S (848561, Advanced Cell Diagnostics, Newark, CA, USA) directed against SARS-CoV-2, targeting the segment between nucleotides 21,631 and 23,303 of the reference sequence. This RNAScope probe is an antisense probe that detects the sense (positive strand). The tumor tissue cores (from before the pandemic) and the non-infected cells were used as negative controls, and a probe to the housekeeping gene peptidylprolyl isomerase B (PPIB–313901, Advanced Cell Diagnostics, Newark, CA, USA) was used to check RNA integrity. The RNAscope 2.5 HD Detection Kit-Brown was used on CMA sections (4 μm) according to the manufacturer’s recommendations. Briefly, InfectionCMAs retrieval was performed at 98 °C for 20 min followed by incubation with RNAscope protease for 30 min at 40 °C. Probes were added and hybridized for 2 h at 40 °C, followed by incubation with AMP1 (30 min at 40 °C), AMP2 (15 min at 40 °C), AMP3 (30 min at 40 °C), AMP4 (15 min at 40 °C), AMP5 (30 min at RT), AMP6 (15 min at RT), and DAB (10 min at RT). Sections were counterstained with hematoxylin, dehydrated and mounted. All incubations at 40 °C were performed in a HybEZ hybridization oven (Advanced Cell Diagnostics, Newark, CA, USA). We confirmed that the tested viral mRNA probe was not expressed on the human tissues (Supplementary Figure S1) and in the mock experiments for Vero and human cells (Supplementary Figure S2).

### 2.8. Western Blotting

Cells were lysed with RIPA buffer (Sigma, St. Louis, MO, USA) supplemented with a protease-inhibitor cocktail (Roche, Basel, Switzerland). Samples were incubated for 30 min at 4 °C. After clearing by centrifugation (17,000× *g*, 15 min), protein concentrations were determined using the DC Protein Assay (Bio Rad, Hercules, CA, USA). Equivalent amounts of protein were separated by electrophoresis in 12.5% SDS-polyacrylamide gels, transferred to nitrocellulose (Amersham Protan, Cytiva, Marlborough, MA, USA) using a wet system (BioRad, Hercules, CA, USA), and analyzed by immunoblotting. The membranes were blocked with 5% BSA and incubated with the specific primary antibodies (the ones used in ICC), and mouse or rabbit HRP-conjugated secondary antibodies. GAPDH (mouse; GTX627408, Genetex, Irvine, CA, USA) and Actin (rabbit; ab8227, Abcam, Cambridge, UK) were used as loading controls. Finally, the membranes were incubated with the Luminata Crescendo Western HRP substrate (Millipore, Burlington, MA, USA), imaged with the ChemiDoc system, and analyzed with associated ImageLab software (Bio-Rad, Hercules, CA, USA). Student’s *t*-tests were applied for pairwise comparisons.

### 2.9. RNA Extraction, cDNA Synthesis and qRT-PCR

In order to evaluate the expression of ACE2 gene, total RNA was isolated with the NZY Total RNA Isolation kit (Nzytech, Lisbon, Portugal), quantified in the NanoDrop 1000 (Thermo Scientific, Waltham, MA, USA), and reverse transcribed to cDNA with the M-MuLV kit (Nzytech, Lisbon, Portugal). The qRT-PCR reaction was performed in triplicate, with the NZYSupreme qPCR Probe Master Mix (Nzytech, Lisbon, Portugal) and predesigned qPCR assay (Hs.PT.58.27645933; IDT, Coralville, IA, USA), run and analyzed in the Applied Biosystems^®^ 7500 Real-Time PCR System (Applied Biosystems, Waltham, MA, USA). TATA-box-binding protein (TBP; no. 4326322E-0705006; Applied Biosystems, Waltham, MA, USA) was used as a housekeeping gene. Data analysis was performed using the 2−ΔΔCT method. Student’s *t*-tests were applied for pairwise comparisons.

## 3. Results

### 3.1. Sequencing of the SARS-CoV-2 Isolate

The sequencing of the isolated viral specimen revealed its affiliation in the B.1.1.29 lineage, with C241T-C3037T-C14408T-T19521C-A20742C-A23403G-G28881A-G28882A-G28883C variants in relation to the reference sequence NC_045512.2. This specimen was common in Europe during the spring of 2020. As this virus was isolated from a hospitalized symptomatic individual, it has pathogenic competence, and could be used in the in vitro infection evaluation. This competence was additionally confirmed through viral titration in each infection assay performed here.

### 3.2. Establishment of an InfectionCMA and Evaluation of Its Efficiency Versus a Traditional Approach

**Figure 1:**
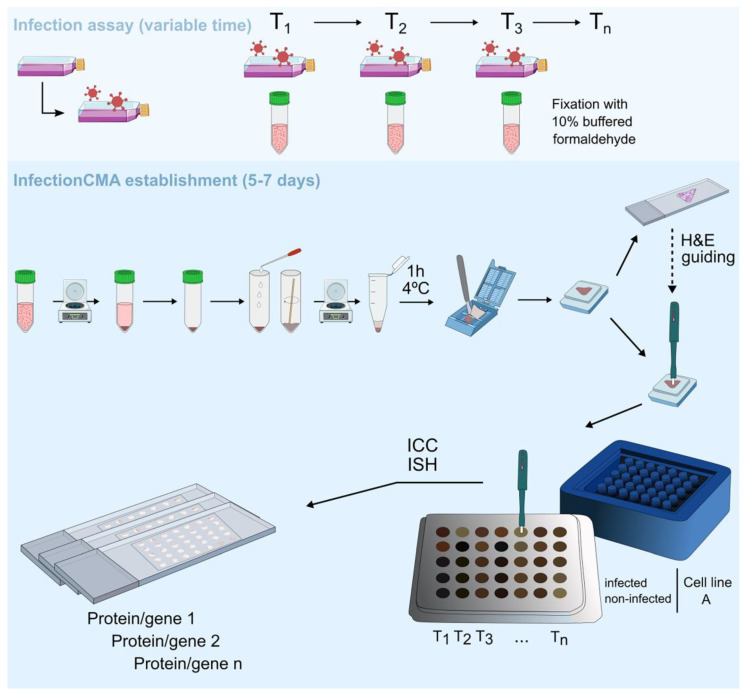
Schematic representation of the procedure to establish the InfectionCMA. The infection assay can have variable timelines depending on the experimental assay. The manual establishment and immune processing of the InfectionCMA take around 5–7 days. A 1–2 cm pellet of infected cells guarantees 30 slides from each InfectionCMA. This biological resource can be used for the expression evaluation of several markers of interest, through diverse techniques such as immunocytochemistry (ICC) and in situ hybridization (ISH).

We decided to illustrate the establishment of an InfectionCMA with the following specific infection kinetic test for SARS-CoV-2: Vero cells infected with the SARS-CoV-2 isolate at two MOIs (MOI 0.01 and MOI 0.001) and control non-infected cells (mock) were grown until four time points after infection (4 h, 24 h, 48 h and 72 h), giving a total of 12 observation-points. These low MOIs are usually used in Vero cells to grow the virus for establishing stocks. The infection assay in BSL3 conditions took three days, and could be done in T-25 flasks, with eight independent infections. After fixation that inactivates SARS-CoV-2, samples were brought out of the BSL3 for the manual establishment of the InfectionCMA and ICC processing, a process that took 5–7 days in total (Figure 1). In a first phase, the cells from the 12 observation points had to be independently processed and included in 12 paraffin blocks; these 12 blocks were sectioned and H&E stained to check morphology and cell orientation in the paraffin blocks; and finally, each of the 12 blocks was individually punched to remove a 1.5 mm-diameter core to build the microarray arrangement in the 5 line × 7 column mold. In a second phase, with the 12 independent points already in a microarray format, the block could be cut in a single slide, for ICC or ISH. This block can be stored for an infinite period of time, and be posteriorly cut several times depending on the initial number of cells. In this specific case, for the initial number of cells used, we could retrieve a 1–2 cm pellet, at each time point, guaranteeing enough amount to obtain 30 slides from the InfectionCMA. This biological resource can be used to evaluate the expression of several markers of interest, specifically 30–90 proteins in total, depending on singleplexing or multiplexing the antibodies (1 to 3 proteins per slide).

**Figure 2:**
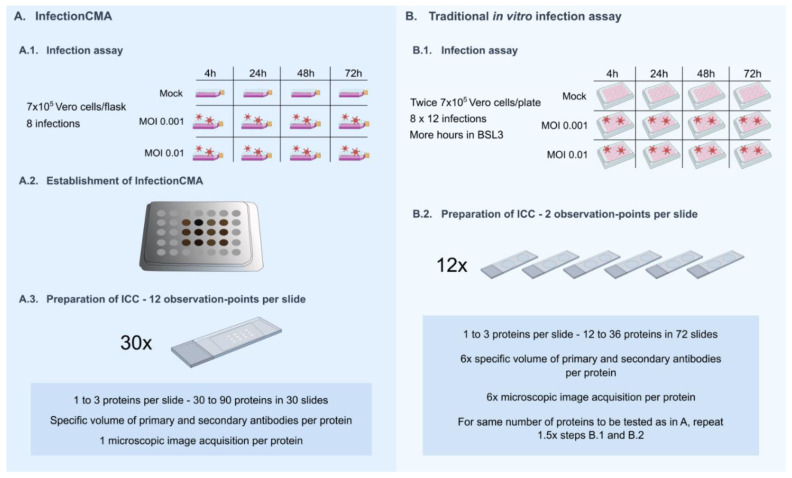
Comparison of the design and outputs between the InfectionCMA and the traditional in vitro infection assay for the first infection kinetics test performed here.

To evaluate the efficiency of the InfectionCMA for this test, in quantitative terms, we estimated the required conditions in a traditional in vitro infection assay in order to obtain identical outputs (Figure 2). Usually, the output from the traditional in vitro infection assay can be improved by increasing the number of cells, viruses, plates and independent infection points, which result in obvious constraints on management, reproducibility, time consumption and used reagents. In the specific case, in order to keep the experiment manageable, we envisioned using double the number of cells and viruses in 12-well plates, implying 8 × 12 independent infections instead of eight. We used 12 independent points each copied 12 times, and all must be processed at the same or within a limited period of time, as the conservation of fixed cells at 4 °C or −20 °C, in several plates, is not practical and is prone to contamination. If we combine the 12 observation points two-by-two in the same slide, we end up with six slides to replicate the InfectionCMA single slide. This implies using a six times greater quantity of primary and secondary antibodies per protein, and also the process to acquire microscopic images multiplies by six. The twelve repetitions imply the simultaneous processing of 72 slides, allowing to ICC only 12 to 36 proteins. In order to obtain the same output of the 30 slides cut from the InfectionCMA, the traditional in vitro infection assay would need to be performed again 1.5×. This would give a final number of 180 slides to be processed.

Note that we only used 12 of the 35 cores available in the 5 × 7 mold for the InfectionCMA. The higher the number of cores used, the higher the efficiency of the InfectionCMA compared with the traditional in vitro infection assay.

### 3.3. Evaluation of SARS-CoV-2 Infection Kinetics in Vero Cells

**Figure 3:**
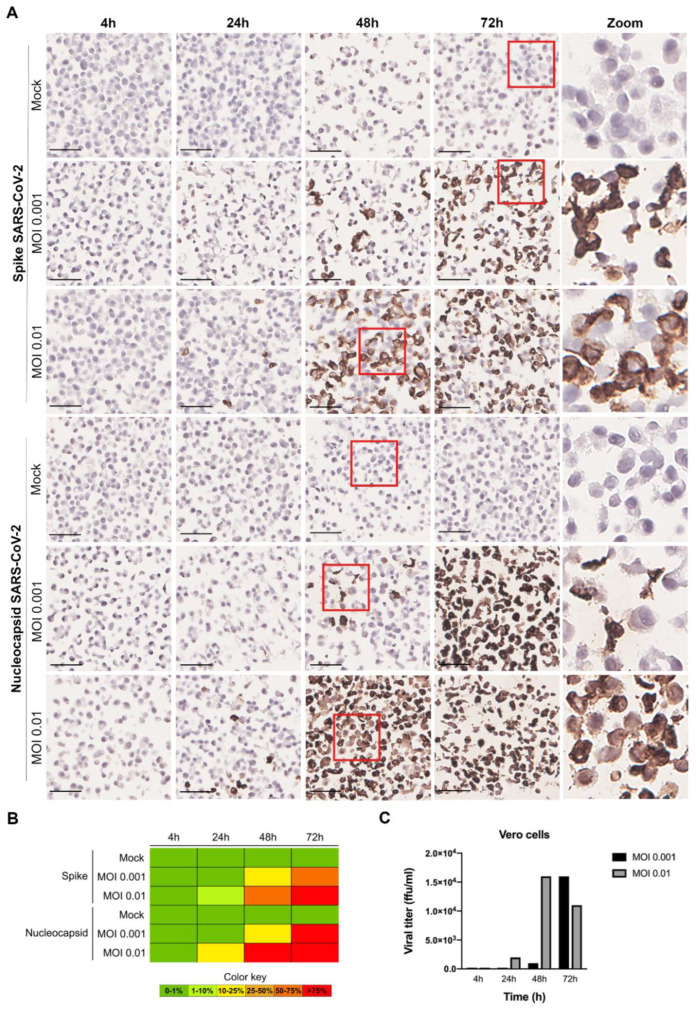
ICC results for SARS-CoV-2 spike and nucleocapsid proteins in infected Vero cells, at various time points (4 h, 24 h, 48 h and 72 h), for MOI 0.001 and MOI 0.01. (**A**) Microscopic images with 50 µm scale bar. The last column represents zoomed-in images of the sections highlighted within a red square in the respective rows. Both viral proteins were located in the cytoplasm of the infected cells. (**B**) Heat map for the percentage of cells stained (0–1%, >1–10%, >10–25%, >25–50%, >50–75%, and >75%). (**C**) Infectious viral particles produced at the various time points (in ffu/mL—focus forming units per mL).

The SARS-CoV-2 infection kinetics in Vero cells was evaluated through the application of ICC for specific SARS-CoV-2 proteins (spike and nucleocapsid) in the InfectionCMA slides (Figure 3). It was possible to observe in the microscopic images that the viral spike and nucleocapsid proteins were located in the cytoplasm of the infected cells. A possible explanation for the signal for the nucleocapsid being stronger than the one for the spike is the former being more abundantly produced than the latter, when the virus is replicating, as observed by others [39]. This can lead to the impression that the nucleocapsid is being produced in more cells than the spike, when in fact it is being detected earlier in cells that are beginning to replicate the virus. At the higher MOI, infection was already visible in the Vero cells at 24 h (between 10 and 20% of infected cells), while at the lower MOI, the same level of infection was only observable at 48 h. Similar time points were observed in other studies [40]. For both MOIs, the rate of infected cells was already higher than 75% around a day later. At the beginning of the infection course, most cells have a low viral load, but by 72 h, most cells have a high viral load, visible by the intense spike staining.

**Figure 4:**
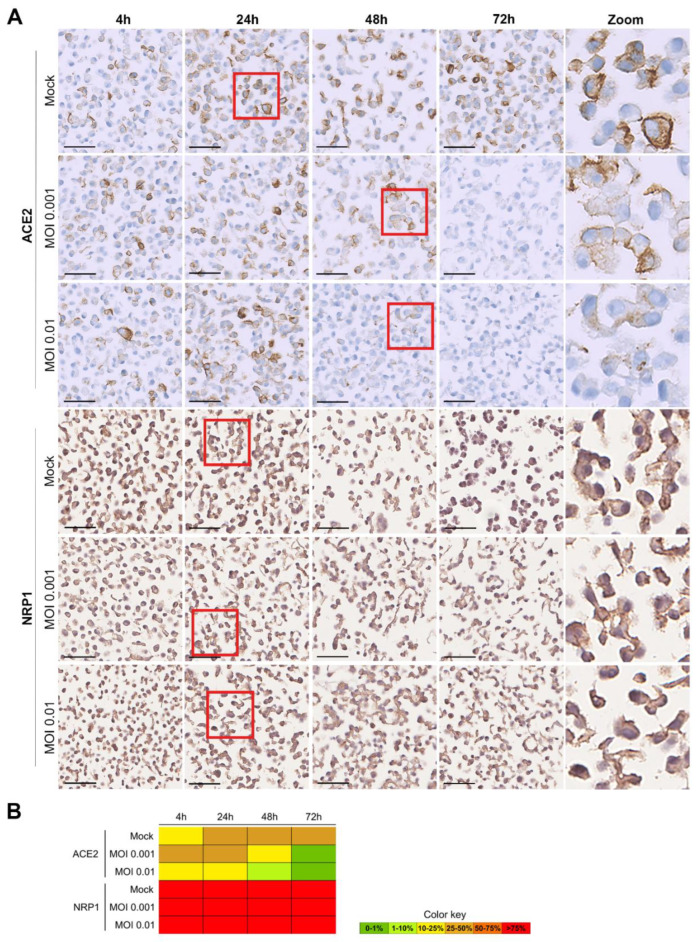
ICC results for ACE2 and NRP1 proteins in SARS-CoV-2-infected Vero cells, at various time points (4 h, 24 h, 48 h and 72 h), for mock (non-infected), MOI 0.001 and MOI 0.01. (**A**) Microscopic images with a 50 µm scale bar. The last column represents zoomed-in images of the sections highlighted within a red square in the respective rows. Both viral proteins were located in the cytoplasm of the infected cells. ACE2 was localized in the membrane, and NRP1 in the membrane and in the cytoplasm. (**B**) Heat map for the percentage of cells stained (0–1%, >1–10%, >10–25%, >25–50%, >50–75%, and >75%).

ICC was also used for the evaluation of the expression of the human receptors involved in SARS-CoV-2 entrance into cells: NRP1 and ACE2 genes. ACE2 was localized in the membrane, and NRP1 in the membrane and in the cytoplasm (Figure 4). The cells were collected by scraping and were not trypsinized, allowing us to keep alterations to membrane receptors as low as possible. A higher percentage of Vero cells were positive for NRP1 (>75%) than for ACE2 (~25%) expression. As already described in the literature [41,42], the expression of ACE2 protein significantly decreased as the SARS-CoV-2 infection progressed. Concordantly with the results for spike and nucleocapsid evaluation, the decrease in the ACE2 protein was visible earlier for the higher MOI (at 24 h–10–25%) than for the lower MOI (at 48 h–10–25%), but at 72 h it was not detected in any of the MOIs. No differences in NRP1 expression in the Vero cells were observable over time in any of the MOIs.

### 3.4. Evaluation of SARS-CoV-2 Infection Kinetics in Human Cell Lines

In order to investigate the SARS-CoV-2 infection kinetics in human cell lines, we established an InfectionCMA for Caco-2 and Huh-7 (in parallel with Vero cells, for comparison). Since Vero cells allow higher viral replication rates due to their lack of genes encoding type I interferons, we increased MOI to 1 in this assay. This was conducted in one experiment for each cell line, with two conditions (mock and MOI 1) for three time points. The resulting InfectionCMA contained 18 observation points, rendering it more efficient than the first kinetic infection test: a total of 30 slides for the InfectionCMA in comparison with 270 slides for the traditional in vitro infection assay; nine times lower quantities of primary and secondary antibodies; and 9 versus 9 × 12 infections.

**Figure 5:**
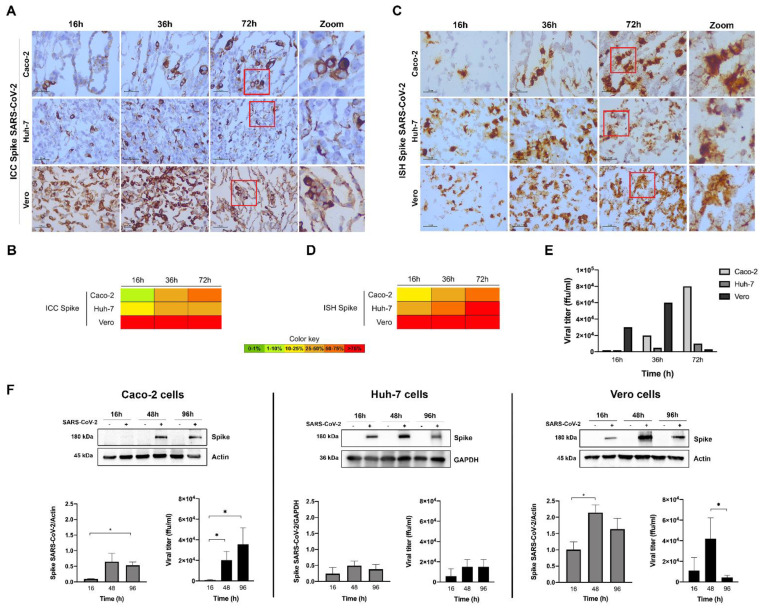
Characterization of SARS-CoV-2 spike expression in the human (Caco-2 and Huh-7) and Vero cell lines, at various time points after infection with MOI 1, by ICC ((**A**) microscopic images with 100 µm scale bar, including zooms of the red inserts; (**B**) heat map), and ISH ((**C**) microscopic images, including zooms of the red inserts; (**D**) heat map). The spike protein had a cytoplasmic location in all the infected cell lines. (**E**) Infectious viral particles produced at the various time points (in ffu/mL—focus forming units per mL). (**F**) Western blots and graphs representing the spike quantification in cells and the infectious viral particles produced in these independent experiments. In the graphs, data represent means ± SD of three independent experiments, and significant Student’s *t*-test *p*-values are indicated (* < 0.05).

Figure 5 shows the results for the quantification of the viral spike protein and mRNA levels. For this high MOI, the ICC results indicate that the Vero cells displayed a high infection rate (>75%) already at 16 h, and the viral titer was higher at 36 h, decreasing significantly at 72 h (Figure 5E; while in Figure 3C, at MOI 0.001, the viral titer was still increasing at this time period, but already decreasing for MOI 0.01). These results show that with a high MOI, the infection rate is very high, and the viral cycle is very fast in the Vero cell lines. Additionally, as ACE2 expression decreases along the infection course, the virions will be less able to reinfect cells, and new replication will begin to decrease (so, decreasing viral titer). In the liver Huh-7 cell line, viral replication was slower (10–25% of cells were positive for the spike protein at 16 h) and showed a steady infection rate of 25–50% along the infection course. In the intestinal Caco-2 cell line, viral replication was even slower (<10% positive cells at 16 h) but reached a higher infection rate at 72 h (50–75%). For both human cell lines, the higher values of viral release to the medium were observed only at 72 h (Figure 5E), testifying the slower infection rate in these cells in relation to Vero cells. These ICC results were broadly supported by the ISH (which is more sensitive than ICC) and the Western blot results for spike, as well as by the ICC results for the nucleocapsid (Supplementary Figure S3). In conclusion, the two human cell lines Huh-7 and Caco-2 are susceptible to infection by SARS-CoV-2 and can be used as in vitro models of infection by this new emergent virus. The evaluation of viral replication kinetics by InfectionCMA and ICC gave reliable results, as compared to well-established techniques such as Western blot and qRT-PCR.

**Figure 6:**
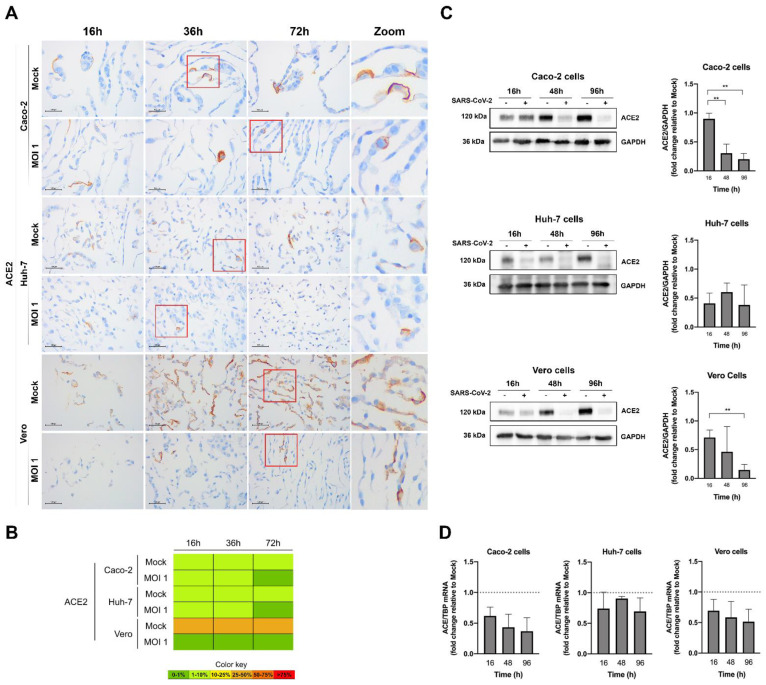
Characterization of ACE2 expression in the human (Caco-2 and Huh-7) and Vero cell lines, at various time points after infection with MOI 1, by ICC ((**A**) microscopic images with 100 µm scale bar, including zooms of the red inserts; (**B**) heat map), Western blot (**C**), and qRT-PCR (**D**). In the graphs, data represent means ± SD of three independent experiments, and significant Student’s *t*-test *p*-values are indicated (** < 0.01). The ACE2 was placed in the membrane of all the infected cell lines.

**Figure 7:**
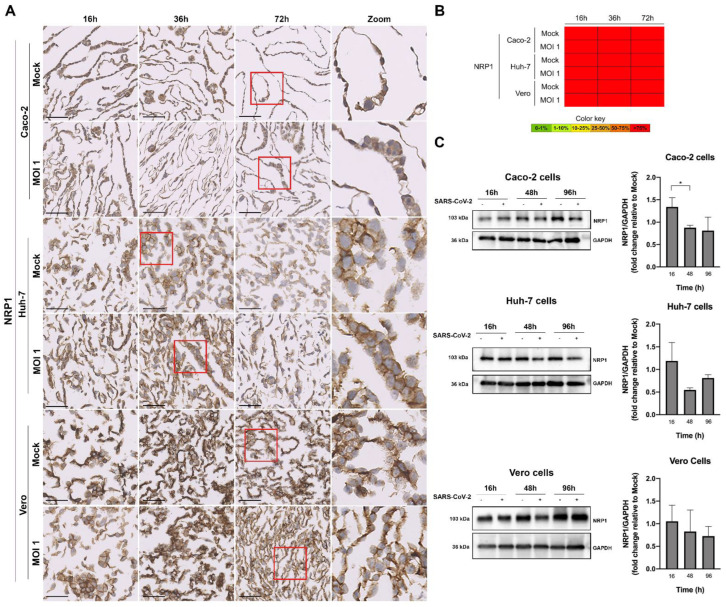
Characterization of NRP1 expression in the human (Caco-2 and Huh-7) and Vero cell lines, at various time points after infection with MOI 1, by ICC ((**A**) microscopic images with 50 µm scale bar, including zooms of the red inserts; (**B**) heat map) and Western blot (**C**). In the graphs, data represent means ± SD of three independent experiments, and significant Student’s *t*-test *p*-values are indicated (* < 0.05). The NRP1 was expressed in the membrane and in the cytoplasm of all the infected cell lines.

Then, we conducted ICC of the human receptors, ACE2 (Figure 6) and NRP1 (Figure 7). NRP1 presented high expression in the non-infected Vero and human cells lines (>75%), while ACE2 proportions was present in 10–25% of all cells. Again, for MOI 1, the decrease in the expression of ACE2 protein as the virus replicated within the human cell lines was clear, being already significant (in relation to the control) at 16 h after infection for the Vero and Huh-7 cells, and only at the time point of 48 h for Caco-2. The decrease with infection was also observable for the ACE2 mRNA when quantified by qRT-PCR. Interestingly, for this higher MOI, NRP1 (Figure 7) followed the same tendency of a decrease in protein expression with the SARS-CoV-2 infection course, but values were overall not statistically significant, probably reflecting the initial higher proportion of NRP1 than ACE2 in all cells.

**Figure 8:**
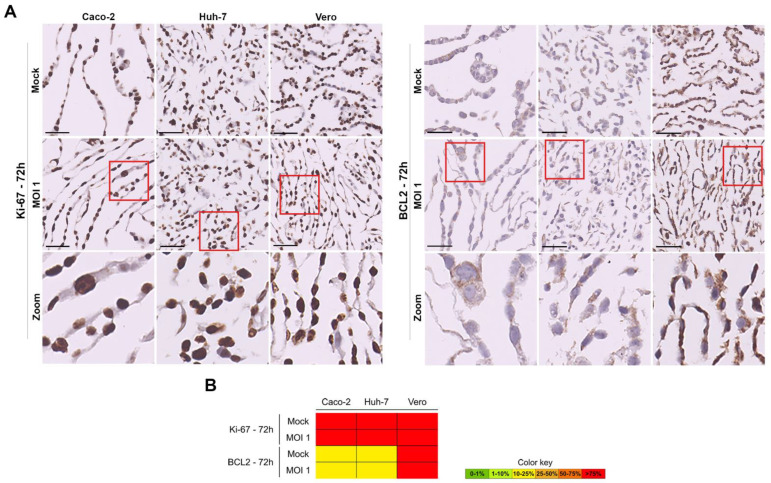
Characterization of Ki-67 and BCL-2 expression in the human (Caco-2 and Huh-7) and Vero cell lines, at 72 h after infection with MOI 1, by ICC ((**A**)—microscopic images with 50 µm scale bar, including zooms of the red inserts in the last row; (**B**)—heat map). The Ki-67 displayed a nuclear and BCL-2 cytoplasmic localizations.

We did not detect visual loss of cell integrity in the course of infection in the Vero and human cell lines. However, in order to evaluate possible alterations in cell viability or proliferation related to viral replication, we performed ICC for two additional markers (Figure 8 and Supplementary Figures S4 and S5): Ki-67, a nuclear protein associated with cellular proliferation [43]; BCL-2 a biomarker of the intrinsic apoptosis pathway [44]. The microscopic images allowed us to confirm the nuclear location of Ki-67 and cytoplasmic localization of BCL-2. There were no statistically significant alterations in proliferation (Ki-67) and in apoptosis (BCL-2) amongst the Vero and human cell lines along the infection course. All cells were homogeneously proliferating, and non-apoptotic (Figure 7 and Supplementary Figures S3 and S4).

## 4. Conclusions

In this work, we illustrate the efficiency of the InfectionCMAs as tools to investigate the infection kinetics of a pathogen, namely SARS-CoV-2. This approach significantly increases the applicability of histology-based methods in virology/infectious diseases field, which traditionally was limited to the viral detection in FFPE samples [45]. The two InfectionCMAs established in this work were easy to implement, and despite not using the maximum output allowed by a 5 × 7 mold, led to substantial gains against a traditional approach: for the 12 observation-point InfectionCMA, it provided 30 slides versus 180 slides and a saving of six times the antibody amounts; for the 18 observation-point InfectionCMA, it provided 30 slides versus 270 slides and a saving of nine times the antibody amounts; and for both, only one compared to 2.5× BSL3 experimental procedures. If the maximum capacity of the 5 × 7 mold was used (an even number of 34 observation-points), the gains would be 30 versus 510 slides and 17 times lower antibody amounts. There are larger molds available, allowing even better outputs. Thus, we demonstrated the added value of InfectionCMA in overcoming difficulties raised by the otherwise complex traditional in vitro infection assays, where improvements in outputs imply increasing the number of cells, viruses, plates and independent infection points. In addition, the InfectionCMA block and slides could be easily stored, and allowed testing of additional markers without the need to conduct a new experiment in biosafety BSL3 conditions. They can also be easily and safely sent to other laboratories, who do not dispose of conditions for viral experiments, for local screening of biomarkers.

Importantly, the evaluation of each marker cytolocalization combined with the morphologic assessment of the infected cell is a powerful tool for a comprehensive histological evaluation of the SARS-CoV-2 infection process and of its key biological players. We confirmed the capacity of SARS-CoV-2 (isolate B.1.1.29) to efficiently infect the monkey Vero and the human Huh-7 and Caco-2 cells. As verified previously [40], Caco-2 were more susceptible than Huh-7. We verified that a significant decrease occurred in the mRNA and protein expressions of ACE2 along the infection course, as observed by others [41,42]. ACE2 is known to be the main human receptor for SARS-CoV-2, and this may explain the fast viral cycle in the permissive Vero cell line at high MOI: the significant decrease in ACE2 expression impaired the reinfection of cells by virions at 72 h (inferred from the decreasing viral titer). However, SARS-CoV-2 can use other host receptors, as demonstrated by Daly et al. [46], when the suppression of NRP1 expression by short hairpin RNA greatly reduced SARS-CoV-2 infection at both 7 h and 16 h after infection in Caco-2 cells. Interestingly, we observed the same tendency of NRP1 protein for decreased expression with infection in the three cell types, when the MOI was high. To the best of our knowledge, this decrease in NRP1 expression with SARS-CoV-2 infection has not been described previously in the literature, probably due to its considerable overall higher expression than ACE2 in most cells and tissues (as can be confirmed in protein atlas and GTEx websites; https://www.proteinatlas.org/ and https://gtexportal.org/home/, accessed on 31 December 2021). This confirmation strengthens the implication that cell types which do not express ACE2 can even so be infected by SARS-CoV-2, either through alternative surface receptors [47] or receptor-independent mechanisms of entry [48]. Additionally, as SARS-CoV-2 acquires further mutations, it can become more efficient in exploring the alternative receptors and mechanisms.

Even at the higher MOI 1, we did not observe significant cell death over the course of infection, for as long as 72 h. Similar observations were reported in comparison studies between SARS-CoV-2 and SARS-CoV infectivity, which found that SARS-CoV-2 replicates to higher levels without inducing substantial host cell damage, while SARS-CoV showed more restricted tropism and higher pathogenicity [40]. This behavior might contribute to prolonged COVID-19 in patients whose immune system is not efficient in fighting SARS-CoV-2.

## Data Availability

The data presented in this study are available in the main text, figures, tables and Supplementary Materials.

## Supplementary Materials

The following are available online at: https://www.mdpi.com/article/10.3390/pathogens11030313/s1, Figure S1: ICC for the spike, nucleocapsid, ACE2, NRP1, Ki-67 and BCL2 in the tumor tissue cores of kidney and liver. Scale bar 50 µm. Figure S2: Characterization of SARS-CoV-2 nucleocapsid expression in the human (Caco-2 and Huh-7) and Vero cell lines, at different time points after infection with MOI 1, by ICC (A–light microscopic images with 100 µm scale bar; B–heat map). Figure S3: Characterization of Ki-67 expression in the human (Caco-2 and Huh-7) and Vero cell lines, at different time points after infection with MOI 1, by ICC (A–light microscopic images with 50 µm scale bar; B–heat map). Figure S4: Characterization of BCL-2 expression in the human (Caco-2 and Huh-7) and Vero cell lines, at different time points after infection with MOI 1, by ICC (A–light microscopic images with 50 µm scale bar; B–heat map).
