# Supplementary material for: InfectionCMA: A Cell MicroArray Approach for Efficient Biomarker Screening in In Vitro Infection Assays
Source: Pathogens. 2022 Mar 3;11(3):313. doi: 10.3390/pathogens11030313 (PMC8955223; doi:10.3390/pathogens11030313)
Supplement: Supplementary file 1 [file pathogens-11-00313-s001.zip › pathogens-1608234-supplementary.pdf]

InfectionCMA: a Cell MicroArray Approach for Efficient Biomarker Screening in *in vitro* Infection Assays

Ana C. Magalhães 1,2,3, Sara Ricardo 1,2,4, Ana C. Moreira 1,3,5, Mariana Nunes 1,2,3, Margarida Tavares 6,7,8, Ricardo J. Pinto 1,2,3, Maria Salomé Gomes 1,3,5 and Luisa Pereira 1,2,*

Supplementary Material:

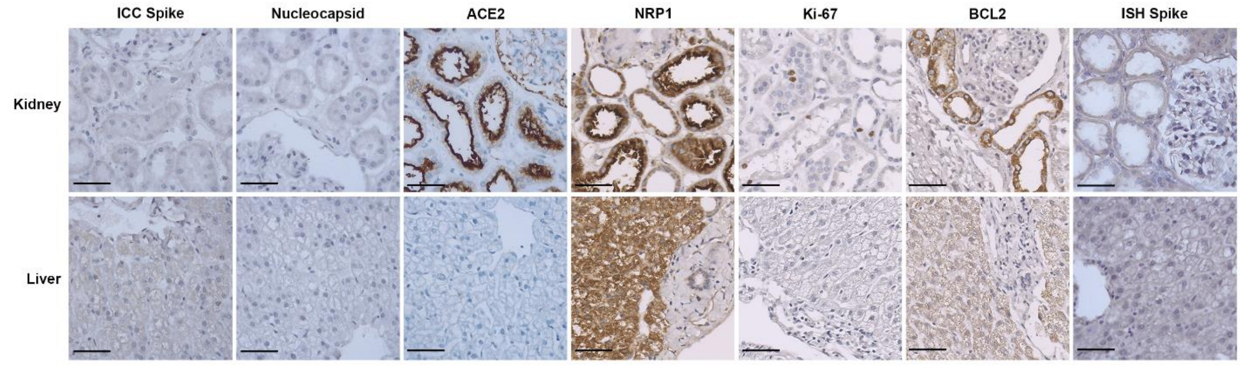

Supplementary Figure S1- ICC for the spike, nucleocapsid, ACE2, NRP1, Ki-67 and BCL2, and ISH for the spike, in the tumour tissue cores of kidney and liver. Scale bar 50µm.

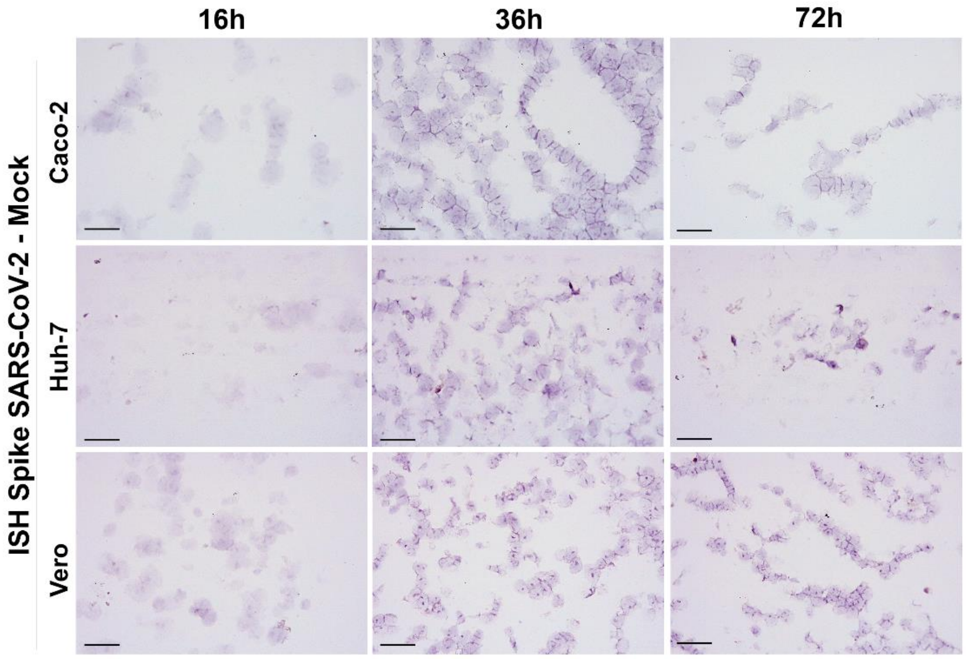

Supplementary Figure S2- Controls for the characterization of SARS-CoV-2 spike mRNA expression in the human (Caco-2 and Huh-7) and Vero cell lines, at different time-points, by ISH. Microscopic images with 100µm scale bar.

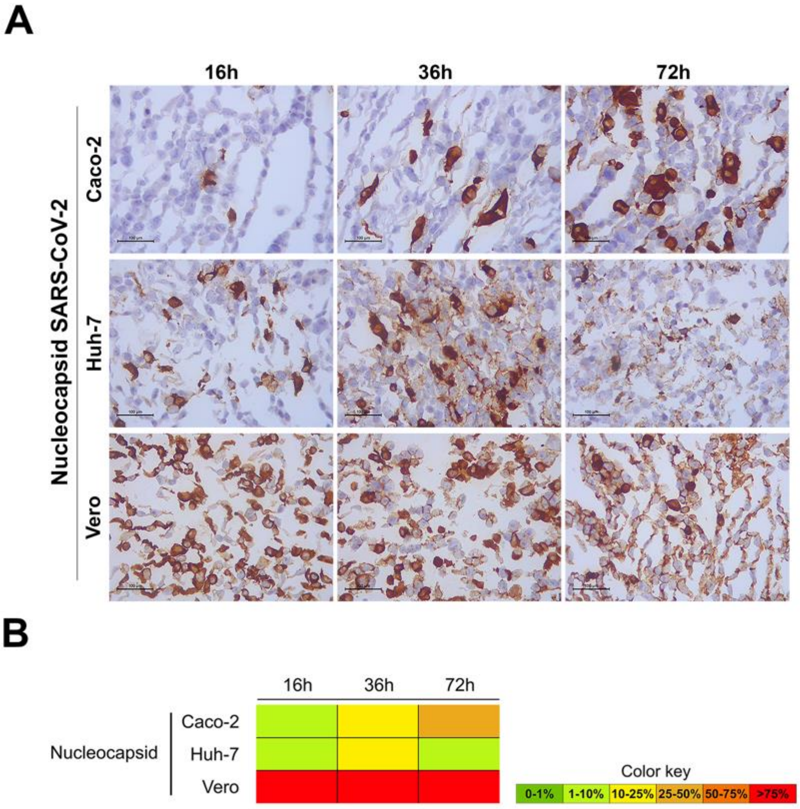

Supplementary Figure S3- Characterization of SARS-CoV-2 nucleocapsid expression in the human (Caco-2 and Huh-7) and Vero cell lines, at different time-points after infection with MOI 1, by ICC (A- light microscopic images with 100µm scale bar; B- heat map).

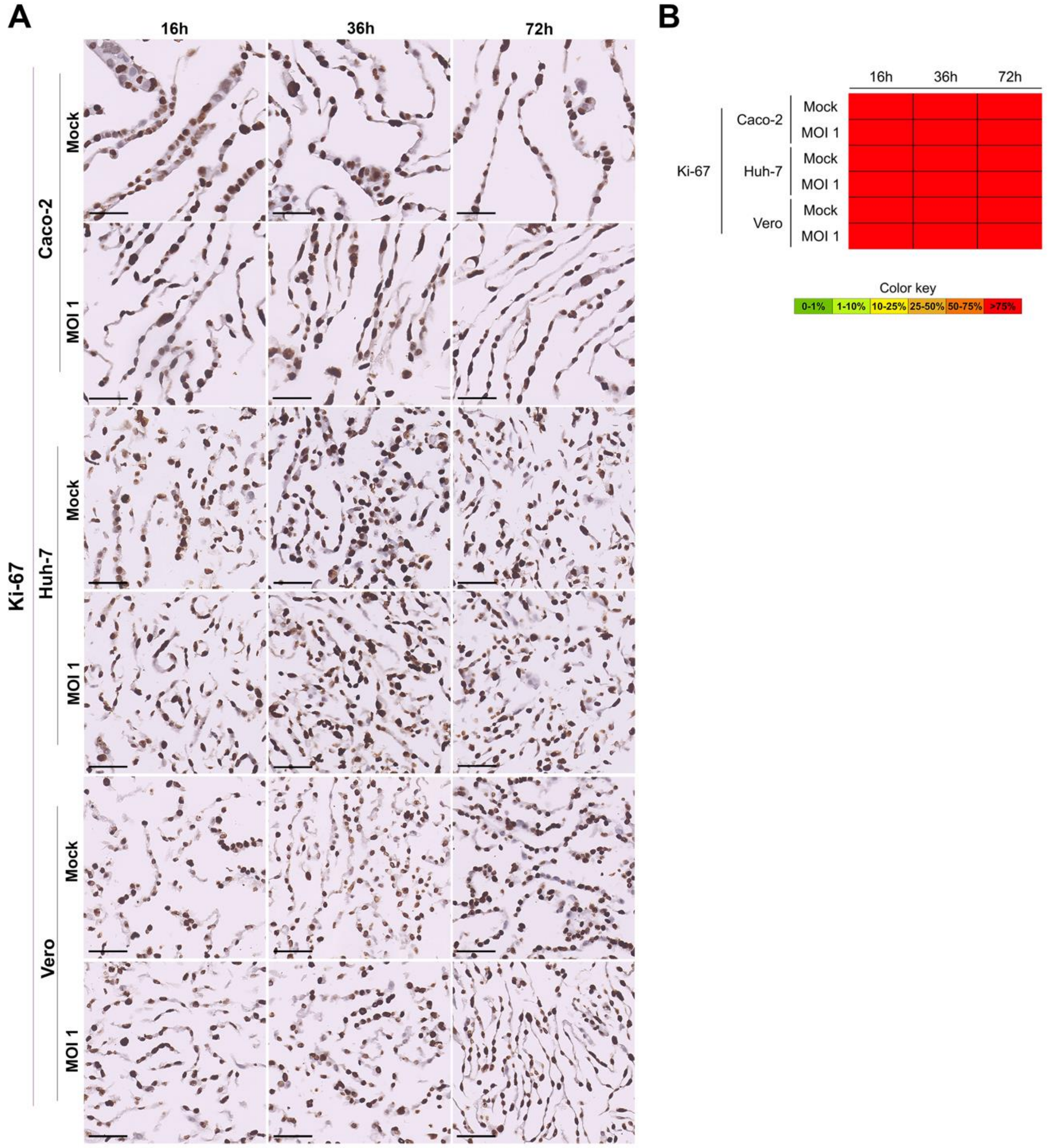

Supplementary Figure S4- Characterization of Ki-67 expression in the human (Caco-2 and Huh-7) and Vero cell lines, at different time-points after infection with MOI 1, by ICC (A- light microscopic images with 50µm scale bar; B- heat map).

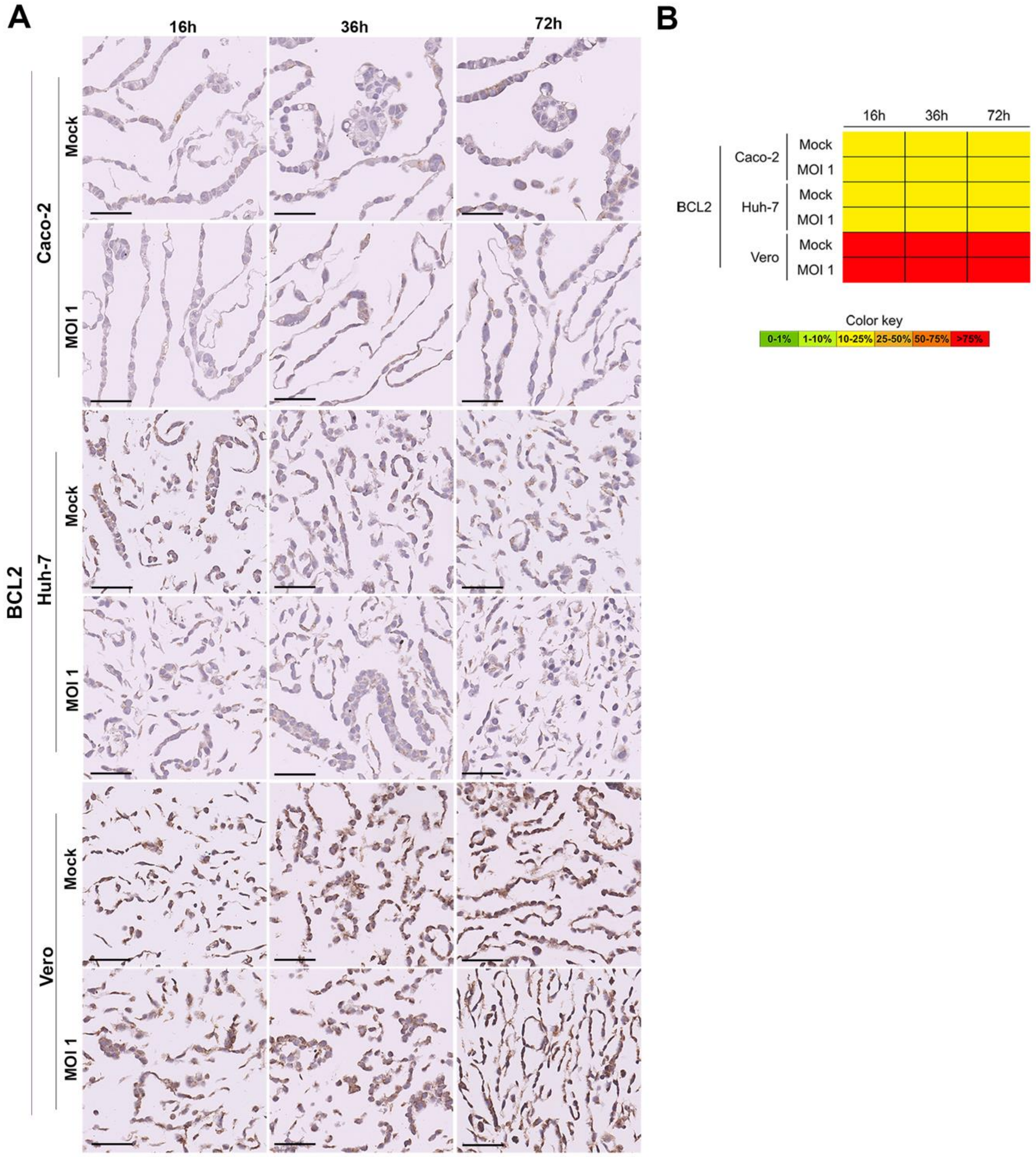

Supplementary Figure S5- Characterization of BCL-2 expression in the human (Caco-2 and Huh-7) and Vero cell lines, at different time-points after infection with MOI 1, by ICC (A- light microscopic images with 50µm scale bar; B- heat map).
